# Impediments to the Success of Management Actions for Species Recovery

**DOI:** 10.1371/journal.pone.0092430

**Published:** 2014-04-03

**Authors:** Chooi Fei Ng, Hugh P. Possingham, Clive A. McAlpine, Deidré L. de Villiers, Harriet J. Preece, Jonathan R. Rhodes

**Affiliations:** 1 School of Mathematics and Physics, The University of Queensland, Brisbane, Queensland, Australia; 2 Australian Research Council Centre of Excellence for Environmental Decisions, The University of Queensland, Brisbane, Queensland, Australia; 3 National Environmental Research Program Environmental Decisions Hub, The University of Queensland, Brisbane, Queensland, Australia; 4 School of Biological Sciences, The University of Queensland, Brisbane, Queensland, Australia; 5 Imperial College London, Department of Life Sciences, Silwood Park, Ascot, Berkshire, England, United Kingdom; 6 School of Geography, Planning and Environmental Management, The University of Queensland, Brisbane, Queensland, Australia; 7 Department of Environment and Heritage Protection, Brisbane, Queensland, Australia; University of KwaZulu-Natal, South Africa

## Abstract

Finding cost-effective management strategies to recover species declining due to multiple threats is challenging, especially when there are limited resources. Recent studies offer insights into how costs and threats can influence the best choice of management actions. However, when implementing management actions in the real-world, a range of impediments to management success often exist that can be driven by social, technological and land-use factors. These impediments may limit the extent to which we can achieve recovery objectives and influence the optimal choice of management actions. Nonetheless, the implications of these impediments are not well understood, especially for recovery planning involving multiple actions. We used decision theory to assess the impact of these types of impediments for allocating resources among recovery actions to mitigate multiple threats. We applied this to a declining koala (*Phascolarctos cinereus*) population threatened by habitat loss, vehicle collisions, dog attacks and disease. We found that the unwillingness of dog owners to restrain their dogs at night (a social impediment), the effectiveness of wildlife crossings to reduce vehicle collisions (a technological impediment) and the unavailability of areas for restoration (a land-use impediment) significantly reduced the effectiveness of our actions. In the presence of these impediments, achieving successful recovery may be unlikely. Further, these impediments influenced the optimal choice of recovery actions, but the extent to which this was true depended on the target koala population growth rate. Given that species recovery is an important strategy for preserving biodiversity, it is critical that we consider how impediments to the success of recovery actions modify our choice of actions. In some cases, it may also be worth considering whether investing in reducing or removing impediments may be a cost-effective course of action.

## Introduction

The majority of the world's species and ecosystems are at risk due to multiple threats [1]–[5]. These threats include habitat loss and fragmentation, invasive species, introduced predators and disease [6], [7]. In addition, climate change is an emerging threat for many species [8]–[10]. Given the limited resources for conservation and the large number of threats, recovery plans must consider how to allocate resources efficiently among actions to mitigate multiple threats.

Allocating resources efficiently requires an understanding of the costs and benefits associated with conservation actions [11], [12]. One way to do this is to identify the highest return-on-investment for our conservation efforts using return-on-investment analysis [13]. This approach is explicit about the costs and benefits of alternative actions and has been used to prioritize actions at a range of spatial scales [14], [15]. The approach also has been applied to understand how to prioritize investment simultaneously across more than one type of action to address multiple threats [10], [16]. A key advantage of the return-on-investment framework in this case is that it allows us to understand the trade-offs inherent in prioritizing the mitigation of different threats. However, although applications of return-on-investment analysis have accounted for the costs and benefits of multiple threat mitigation actions, in general, they have failed to consider how impediments (or constraints) to the success of each action drives conservation priorities.

Most conservation, or recovery, actions are subjected to a range of impediments to their success [17]–[22]. In considering this, the conservation planning literature has mainly focused on the social factors that drive opportunities (and constraints) for the implementation of conservation actions [20], [22]. As a result of this we have, for example, a reasonably good understanding of how landholders' willingness to sell their land affects conservation priorities [23], [24]. However, the role of impediments to the success of conservation actions can be complicated by the fact that different actions may be subjected to different types or sizes of impediments. For example, Prugh et al. [25] find that, for threatened species in Canada, the political ease with which different threats can be mitigated varies among threats depending on the industry causing the threat. There may often also be a wide range of different causes of impediments to success, ranging from socio-economic to logistical and technological factors [26]. In these cases, the optimal investment in recovery actions will depend on the interaction between the costs and benefits of actions to mitigate different threats, but also, importantly, on the relative size and types of the impediments to their success. An explicit consideration of these impediments is therefore critical for the prioritization of actions to mitigate multiple threats, but this has received very little attention to date.

In this paper, we address this issue by examining to what extent different impediments to success drive investment in recovery actions to mitigate multiple threats. We first describe a decision framework to allocate resources optimally among multiple threats to recover a declining population. We then apply this to a declining koala (*Phascolarctos cinereus*) population in eastern Australia, threatened by vehicle collisions, dog attacks and habitat loss and assess the extent to which the allocation of resource among management actions are driven by different impediments to success that include: the unwillingness of dog owners to restrain their dogs at night, the effectiveness of wildlife crossings to reduce vehicle collisions, and the unavailability of areas for habitat restoration. We show that these impediments severely limit our ability to achieve recovery of the population, but also affect the optimal allocation of resources among recovery actions. This illustrates the importance of explicitly considering impediments to success in designing species' recovery plans.

## Methods

### Study species and region

The koala is an arboreal folivorous marsupial primarily restricted to the eucalyptus forests of eastern and southern Australia and is an iconic species of conservation concern in Australia [41]. One of the largest koala populations occurs in the Koala Coast, located 20 km southeast of Brisbane, Australia (area 375 km^2^, human population size 2 million). Rapid urbanization has resulted in habitat loss and fragmentation in the area that has also increased koala mortality rates due to vehicle collisions, dog attacks and disease [41]–[43]. As a result, the koala population has declined by 64% over the past 10 years, from an estimate of 6,250 individuals in 1996–1999, to an estimate of 1,990 individuals in 2010 [44]. Identifying cost-effective strategies for the recovery of this population is a priority.

### The decision problem

To first identify priorities for the recovery of this population, we applied a decision theoretic framework to decide how to allocate resources among various actions to mitigate multiple threats. This framework includes: (1) a management objective, (2) a list of management actions and the costs of implementing these actions, (3) a model of how these actions affect the population dynamics and abundance, and (4) an algorithm to find the optimal management strategy [45].

To formulate the decision framework, let **x** be an *n* x 1 control vector representing the amount of money invested in each of *n* actions, with *x_m_* representing the elements of the vector **x**. Also let λ(**x**) be the growth rate of the population (that depends on the investment in each action **x**) and *R* be a population growth rate that we want to achieve (which we subsequently refer to as the target growth rate). We assume that our management objective is to find the optimal investment strategy that attains the target growth rate for minimum cost,
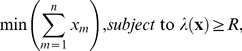
(1)


### Population dynamics

For the Koala Coast koala population, the population growth rate, λ, was obtained based on an existing age-structured matrix model [42] by calculating the dominant eigenvalue of the projection matrix of that model [46]. The matrix model assumes four age classes: juveniles (0–1 year olds), sub-adults 1 (1–2 year olds), sub-adults 2 (2–3 year olds) and adults (3+ year olds) [47], with projection matrix,
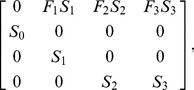
(2)where S*_i_* is the probability of individuals surviving age class *i* and *F_i_* is the probability of individuals giving birth in age class *i*. The model is a female-only model because females are the limiting sex and assumes a discrete breeding season, which occurs from October to February each year [47]. Note that the probability of individuals giving birth, *F_i_*, is multiplied by the probability of survival, *S_i_*, in the first row of the projection matrix because juvenile koalas are dependent on their mother for approximately the first year of their lives and so we assumed that the death of a mother also results in the death of any dependent young [48].

The model also incorporates cause-specific mortality rates based on the key threats, such that, the probability of survival is,
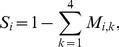
(3)where *M_i,k_* is the mortality probability due to cause *k* for age class *i*. The causes of mortality present in the study region and incorporated into the model are: natural (*k* = 1), vehicle collision (*k* = 2), dog attack (*k* = 3), and disease (*k* = 4).

The mortality probability due to cause *k* for age class *i* can be written as,
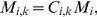
(4)where *C_i,k_* is the probability that, given a mortality event, it arises due to cause *k* for age class *i* and *M_i_* is the unconditional mortality probability for age class *i*. The probability that, given a mortality event, it arises due to cause *k* for age class *i* is related to the forest cover as follows,
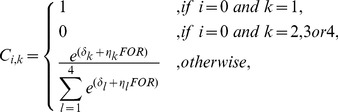
(5)where δ*_k_* is an intercept for cause *k*; *FOR* is the amount of forest cover surrounding the location at which mortality is estimated; and η_k_ is a coefficient that describes the influence of forest cover on *C_i,k_* [42]. Note that juvenile koalas (age class 0) were assumed to only die of natural causes. This equation relates forest cover to the probability that a mortality event arises from each of the causes and is based on a standard logit transformation for a multinomial distribution.

The unconditional mortality probability for age class *i* was also assumed to depend on the amount of forest cover surrounding a location, as follows, 
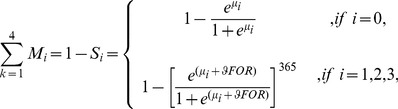
(6)where *µ_i_* is an intercept for the survival probability for age class *i* and *φ* is the coefficient that describes the influence of forest cover on survival probability [42]. Here, adult survival is represented as a daily probability and then annualised, while juvenile survival is modeled directly as an annual rate because data only on annual survival for juveniles was available.

Substituting equations 5 and 6 into equation 4 gives the mortality probability due to cause *k* for age class *i* as,
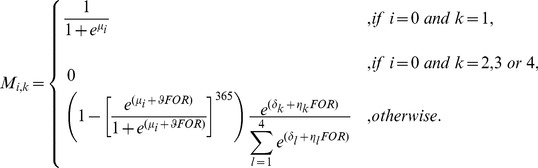
(7)


It was assumed that the amount of forest cover has little influence on birth rates [42]. As such, the population growth rate depends upon the level of mortality from each cause and the amount of forest cover.

Rhodes et al. [42] provide estimates of the parameters of the model derived within a Bayesian framework based on radio-tracking and population density data. We used approximations of the posterior distributions of these parameters based on 9,900 draws from their posterior distributions derived using Markov Chain Monte Carlo methods (MCMC) in WinBUGS Version 1.4.3 (http://www.mrc-bsu.cam.ac.uk/bugs/).

### Return-on-investment curves

To obtain a functional relationship between the population growth rate, λ(**x**), and the level of investment in recovery actions, **x**, we considered three possible actions that could be used to reduce mortality rates: 1) actions to reduce dog attacks, 2) actions to reduce vehicle collisions, and 3) habitat restoration. Actions to reduce dog attacks were assumed to consist of restraining domestic dogs at night in residential areas (Daniel Carter, personal communication). Actions to reduce vehicle collisions were assumed to consist of the construction of wildlife crossings, including underpasses and overpasses, with fencing [49]. Habitat restoration was assumed to involve the setting aside of land for conservation and active restoration of koala habitat. We developed functional relationships between the costs of these actions and the cause-specific mortality rates (which we subsequently refer to as the “return-on-investment-curves”) that allowed us to characterize the relationship between investment in each action and survival and therefore also the population growth based on the dominant eigenvalue of the projection matrix.

We assumed that the survival probability for age class *i* after an investment *x_m_* in action *m* is
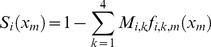
(8)where *f_i,k,m_(x_m_)* is a function that describes how an investment of *x_m_* in action *m* reduces the probability of koalas of age class *i* dying due to cause *k* (a value of one indicates there has been no reduction in mortality and a value of zero indicates that mortality from that cause has been completely eliminated). For these return-on-investment curves, we assumed diminishing marginal returns with increasing levels of investment in each action [13]. In the absence of prior information to motivate a more complex relationship, the functions *f_i,k,m_*(*x_m_*) were described by a negative exponential function, such that 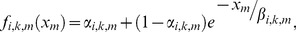
(9)where α*_i,k,m_* is an asymptote between 0 and 1 that describes the minimum value for *f_i,k,m_(x_m_)* and 1/β*_i,k,m_* represents the rate of decline in *f_i,k,m_(x_m_)* as we invest in action *m* (i.e., the cost efficiency of action *m*).

### Parameterising return-on-investment curves with and without impediments to success

We first parameterised the return-on-investment curves (equation 9) for each of the three actions (dog control, reducing vehicle collisions, and habitat restoration) assuming there are no impediments to success using empirical data on the costs of each action obtained for the Koala Coast (see Appendix S1). In this case, we assumed that α*_i,k,m_* = 0 for the dog control and vehicle collision reduction actions (assuming they can reduce dog and vehicle collision mortalities to zero with sufficient investment), but estimated α*_i,k,m_* from simulations for habitat restoration (where we assume that the replacement of habitat reduces natural and disease mortality [42]. We then modified each of the return-on-investment curves to account for impediments to success associated with each action. We achieved this by modifying the value for α*_i,k,m_* to reflect the impact of each impediment on the maximum possible reduction in mortality. For each of the actions, the impediments that limit the success of the action are different and we explicitly accounted for these differences within our framework.

For dog control, there exist human social impediment that arise due to the unwillingness of some dog owners to enclose their dogs at night. Using data from Clark [50], we incorporated the influence of this impediment into the return-on-investment curves. Clark (2006) show that 56% of dog owners who currently keep their dogs outside at night are unwilling to adopt enclosures under any circumstances. We therefore assumed that, due to this social impediment, we can only ever reduce the mortality rate due to dog attacks by up to a maximum of 44%. Consequently, under this impediment for the dog control action, we fixed *α_i,3,dog_* = 0.44 in equation 9 and then re-estimated the other parameters (see Appendix S1).

Existing technology for preventing mortalities on roads that involves the building of wildlife crossings and fencing is generally not capable of eliminating road mortalities entirely [51]. This is partly because we are uncertain about which technologies are best for which species [51], [52]. But, it is also because existing technologies are usually not completely effective in stopping movement across the road surface, for example, due to the permeability of fencing [53]. In the absence of data from the Koala Coast on the effectiveness of road crossings, we used data on koala mortalities from the Bonville upgrade of the Pacific Highway, New South Wales, Australia [54] to characterize the implications of these technological impediments. Semeniuk et al. [54] show that the overpasses and fencing in this location reduced koala road mortalities by 77%. Therefore, we assumed that the vehicle collision mitigation measures could only reduce road mortalities by a maximum of 77% and so fixed *α_i,2,car_* = 0.23 in equation 9 and then re-estimated the other parameters (see Appendix S1).

For habitat restoration, urban and other intensive land-uses will impede implementation because they are unlikely to be available for restoration, or to allow successful restoration because they are highly modified. We accounted for these land-use impediments by re-estimating the parameters of equation 9, assuming that urban and other intensive land-uses are unavailable for restoration (see Appendix S1).

### Solving the decision problem

We found the optimal allocation of resources for target growth rates, *R*, between 0.935 (the current estimated growth rate) and 1.03 (the estimated maximum achievable growth rate), for both with and without the impediments to success. To find the optimal allocation of resources, we used an active set algorithm available via the ‘fmincon’ nonlinear constrained optimization function in Matlab Version R2010a (Mathworks 1984–2010). The active set algorithm uses Lagrange multipliers to calculate the optimal investment in each action that satisfies the management objective (see Appendix S2). We calculated the optimal strategy for each target growth rate based on the posterior mean of the population model parameters and then calculated the 95% credible interval for the growth rate for each target based on the parameter posterior distributions. Finally, we compared the optimal strategy and growth rate achieved when impediments to success are ignored, with when impediments to success are accounted for.

## Results

**Figure 1:**
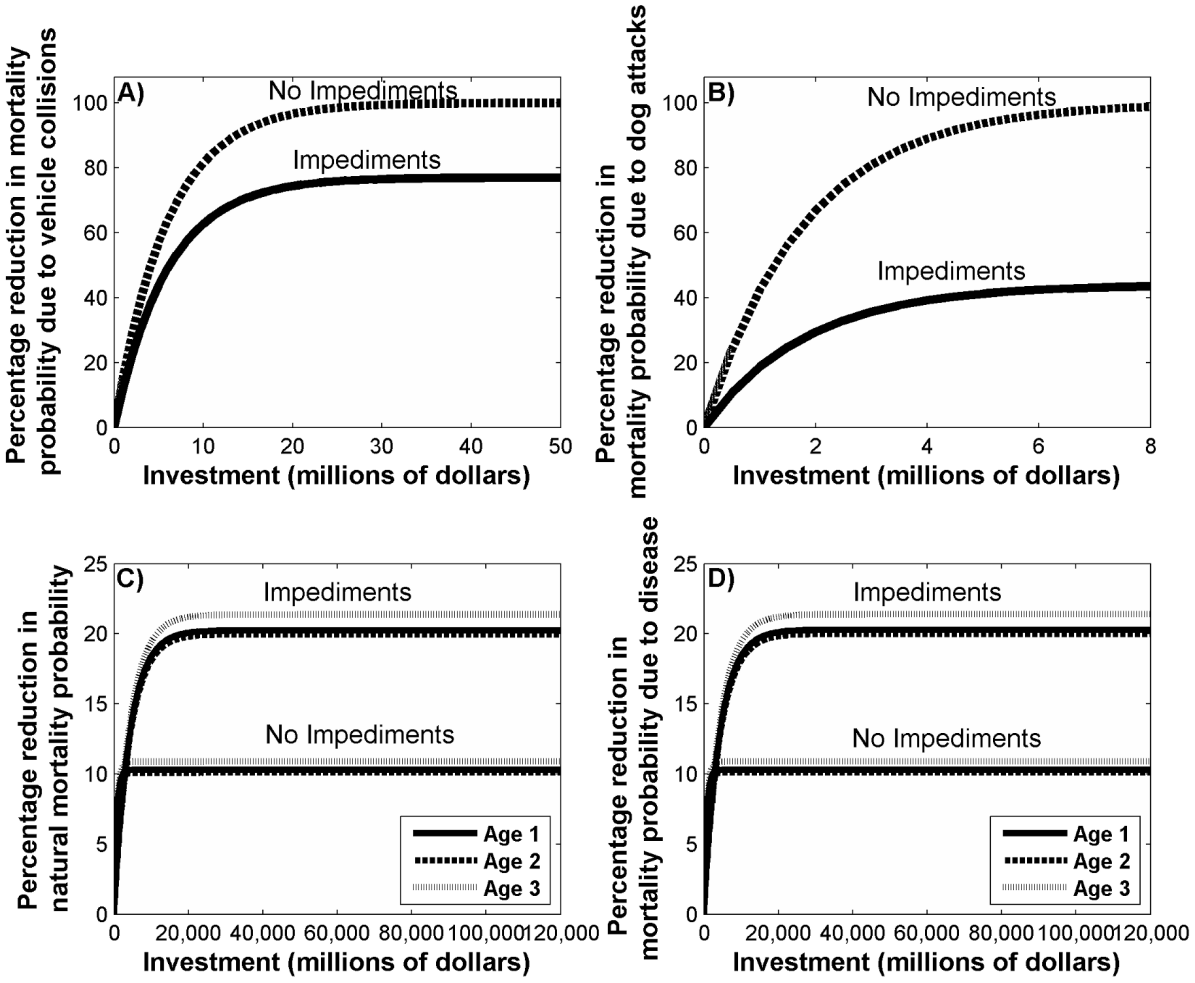
Percentage reduction in the mortality probability with and without impediments to success. When the investment is to (a) reduce vehicle collision mortality; (b) reduce dog attack mortality; (c) reduce natural mortality through habitat restoration; and (d) reduce disease mortality through habitat restoration. For (a) and (b) the relationship was the same for all age classes, except juveniles, and in (c) and (d) the dashed line applies to 1–2 year olds (age class 1), the solid line applies to 2–3 year olds (age class 2) and the dotted line applies to 3+ year olds (age class 3).

Overall investment in dog control and reducing vehicle collisions was considerably more cost-effective than habitat restoration (Fig. 1). For each management action, impediments limit the success of the action. For dog control, there exists a social impediment due to the unwillingness of dog owners to restrain their dogs at night. When mitigating vehicle collision, there exists a technological impediment that reduces the effectiveness of wildlife crossings. For habitat restoration, land-use will impede implementation because urban and other intensive land-uses are unavailable for restoration. When impediments to success were included, the effectiveness of our management actions was substantially reduced, although the extent to which this was true varied among actions (Fig. 1). Among the management actions, introducing impediments had the greatest impact on the effectiveness of dog control (54% reduction in effectiveness), followed by habitat restoration (50% reduction in effectiveness), and the smallest impact was on mitigating vehicle collisions (23% reduction in effectiveness). The return-on-investment curves for habitat restoration among the different age classes had qualitatively similar results for both natural and disease mortality (Fig. 1c & 1d).

**Figure 2:**
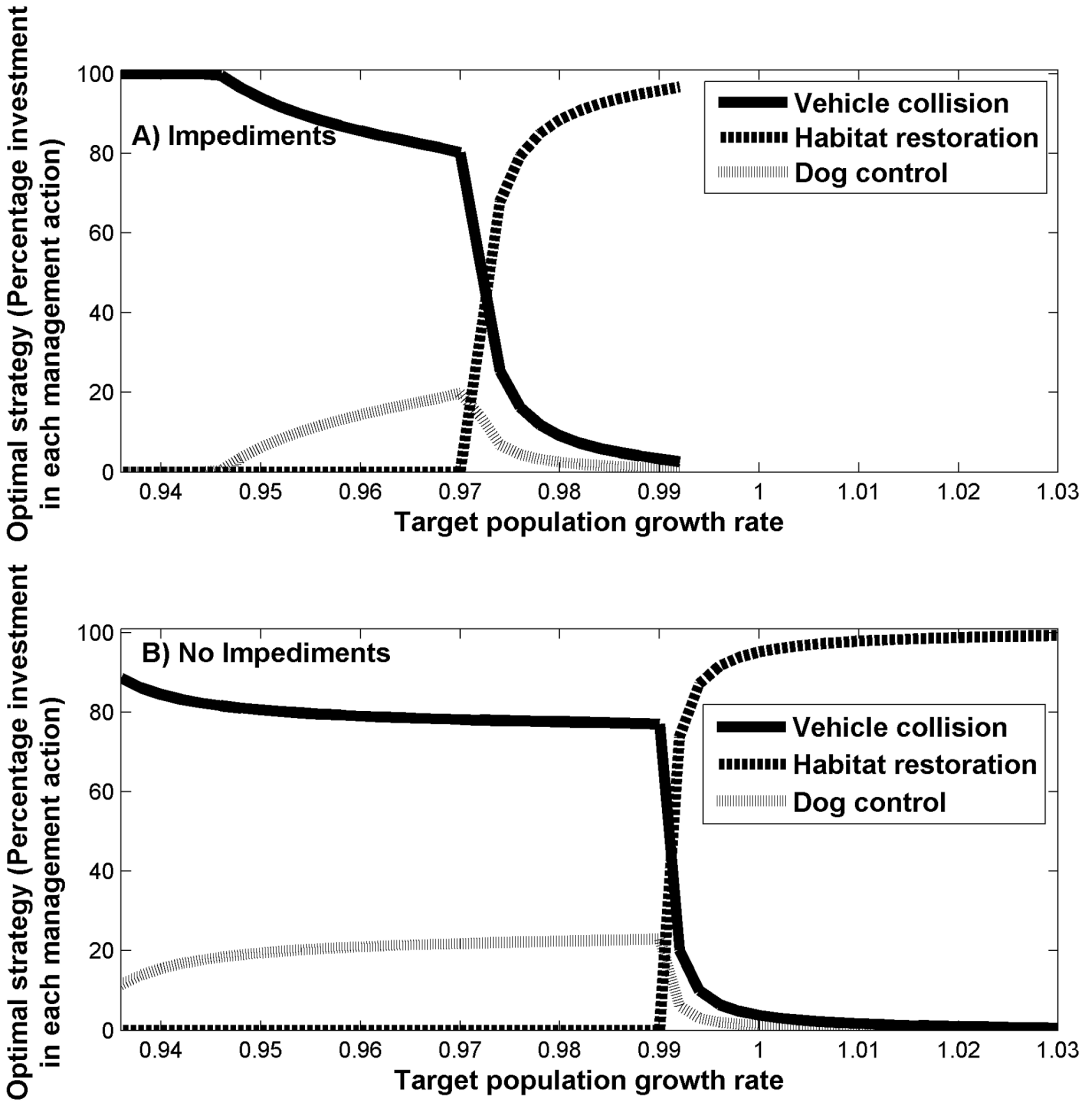
The optimal management strategy (percentage investment in each management action) to achieve the target population growth rate. When we (a) include and (b) exclude impediments to success (the solid line corresponds to reducing vehicle collisions, the dashed line corresponds to habitat restoration, and the dotted line corresponds to dog control.

The optimal strategy for resource allocation among the management actions depended on the target population growth rate (Fig. 2). To achieve a low target growth rate (i.e., up to 0.97 with impediments and up to 0.99 without impediments), the optimal strategy was to invest predominantly in vehicle collision mitigation and dog control measures. However, to achieve target growth rates higher than this, the optimal strategy shifts rapidly towards habitat restoration (Fig. 2). Importantly, however, with the impediments, this shift occurs at a much lower growth rate (0.97) than without the impediments (0.99). Therefore, for target growth rates between 0.97 and 0.99, the optimal strategies with and without impediments are substantially different. In addition, the maximum possible population growth rate that can be obtained was lower in the presence of impediments (0.99) than in their absence (1.03).

**Figure 3:**
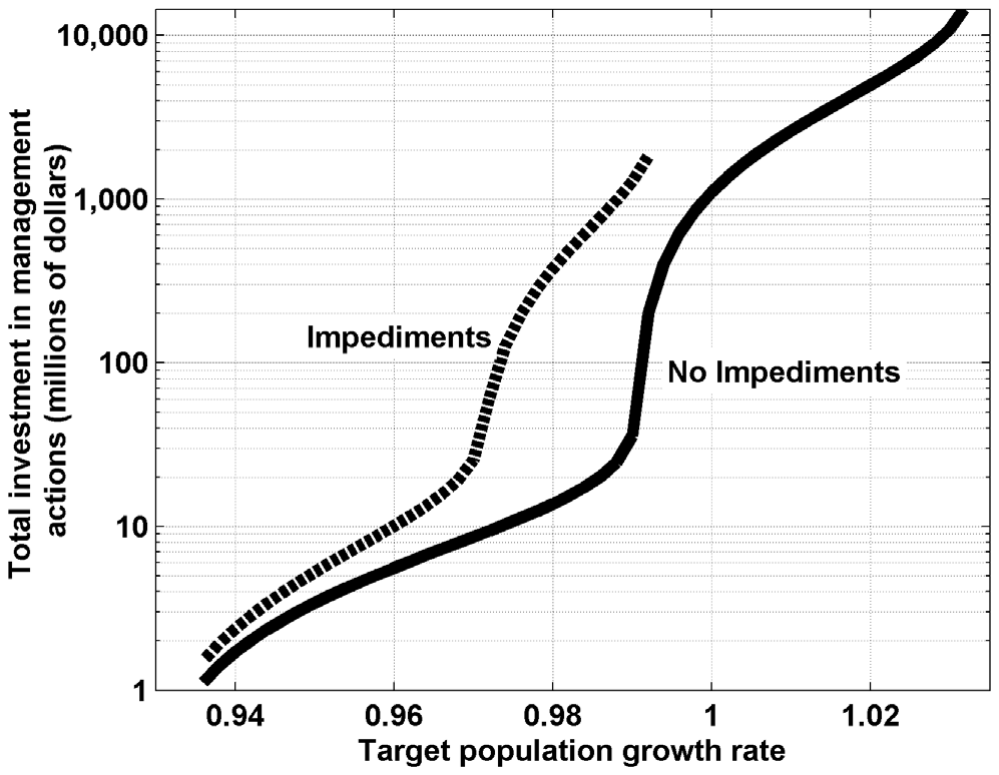
The total investment required to attain the target growth rate. When we include (dashed line) and exclude (solid line) impediments to the success of the actions.

**Figure 4:**
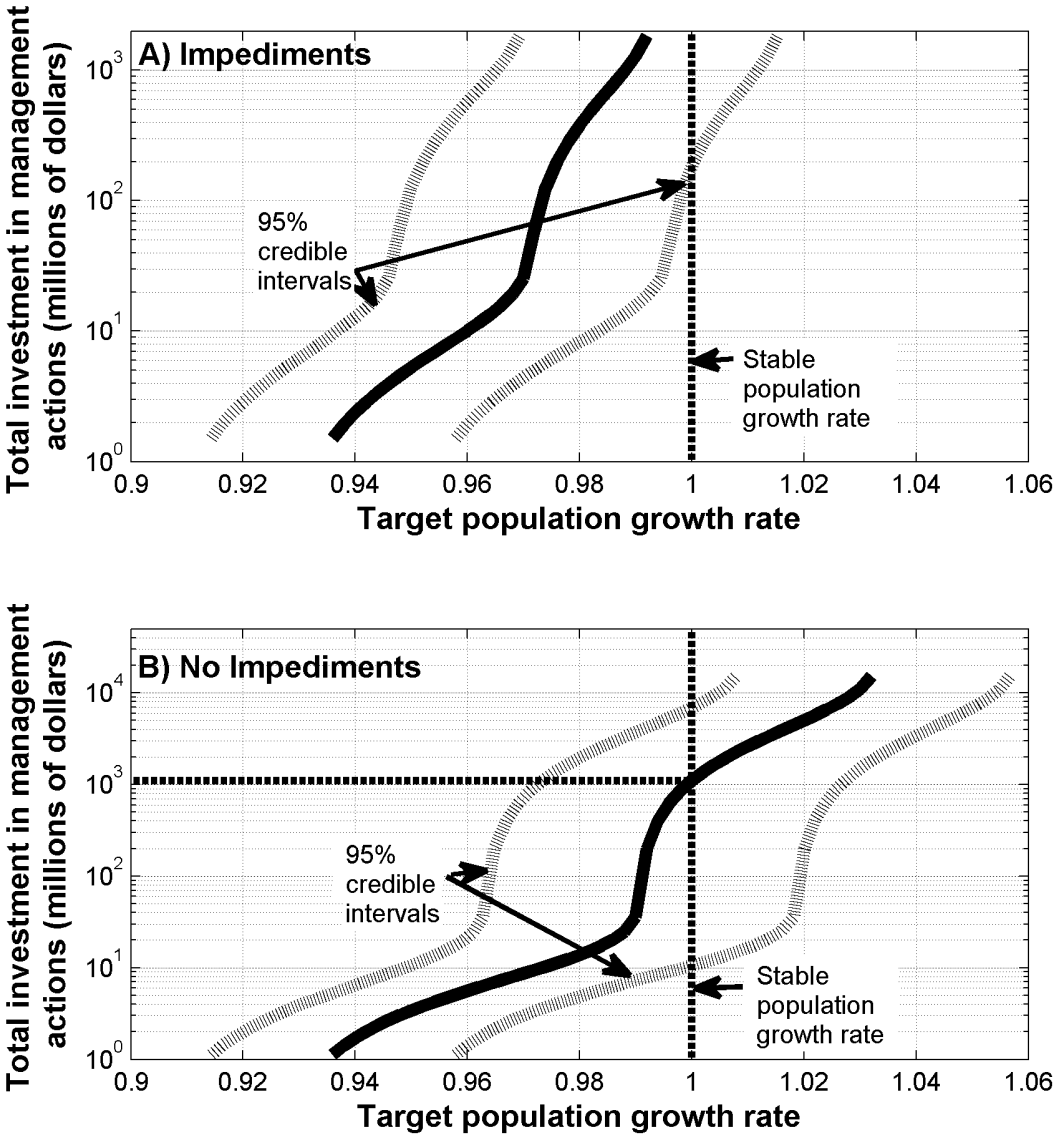
The total investment required to attain the target growth rate, with 95% credible intervals. When we (a) include and (b) exclude impediments to the success of the actions (the dotted line indicates the point-wise 95% credible intervals for the growth rate and the horizontal dashed line indicates the total investment required to achieve a stable population size, i.e., λ = 1).

As we increased the target growth rate, the total investment required increases (Fig. 3). This increase in required investment is very rapid at the point at which the optimal strategy shifts toward habitat restoration (i.e., at a growth rate of around 0.97 when impediments are present and a growth rate of 0.99 when they are absent) (Fig. 3). Therefore, a low growth rate can be achieved relatively cheaply, but achieving growth rates closer to one is considerably more expensive. However, for a given target growth rate, the level of investment required was also considerably higher when we incorporated impediments to the success of the actions than when we did not (Fig. 3). For instance, a population growth rate of 0.97 can be obtained for an investment of AU$25 million in the presence of impediments, but only AU$9 million when they are not present (Fig. 3). However, since the growth rate that we will actually achieve is uncertain, the actual investment required to attain these growth rates is also uncertain (Figure 4). When we take into account uncertainty in parameter estimates, the estimated level of investment required to achieve a population growth rate of 0.97 ranges from around AU$3 million to around AU$1 billion with a 95% probability in the presence of impediments, but from around AU$1 million to around AU$100 million with a 95% probability in the absence of impediments. The presence of impediments in this case also increases the level of uncertainty at the upper end of the estimates of investment required.

## Discussion

We have presented an approach for finding the optimal strategy to invest in actions to mitigate multiple threats simultaneously, while taking account of a range of impediments to the success of each action. Importantly, it allowed us to explore the implications of those impediments for the optimal allocation of resources and conservation outcomes. This is a significant advance because these types of impediments have rarely been considered in conservation decision making for recovery planning [19], [27]. We found that impediments to success severely limits our ability to achieve species recovery and has an impact on the optimal investment in each management action for some recovery targets. In general, given that actions to achieve species' recovery are often subject to a wide range of different impediments [25], this demonstrates that it is critical to carefully consider the impediments to success for each management action in recovery planning.

Impediments to the success of management actions can reduce our ability to achieve our management objectives across a wide range of circumstances. For instance, socio-economic impediments, such as the willingness of land owners to sell their land can reduce the ability to achieve conservation targets as we expand a reserve network [22], [24]. In particular, these impediments can increase the difficulty of meeting targets when allocating resources spatially [22], [24]. Impediments to success, other than those caused by social factors, have received little attention in the literature. However, our work demonstrates that other types of impediments such as those caused by technology and land-use can be equally important. The combination of the different types of impediments made it very difficult to achieve population recovery (i.e., λ>1) in our koala case study. In extreme cases where impediments cannot be avoided, it may be necessary to consider shifting resources elsewhere, rather than wasting scarce resources when the recovery of a species is unlikely [28], [29]. However, in making decisions such as this, careful consideration of the impediments and their consequences for recovery success is critical.

One possible strategy in the face of impediments to success is to remove the impediments that reduce the effectiveness of management actions. However, from a cost efficiency perspective, this requires information about the costs and benefits of reducing the impediments compared to the costs and benefits of managing threats. For example, it may be possible to reduce social impediments to the success of conservation programs through adaptive co-management [30], but the costs and benefits of such an approach relative to top-down conservation management actions have rarely been explicitly assessed. For our koala case study, a possible solution to reducing the impediment to dog attack mortalities may be to use education programs to promote responsible dog ownership and the importance of enclosing dogs at night to protect wildlife [31]. Another strong impediment to the successful reduction of disease mortality is the limited technology to treat disease in koalas directly, such as chlamydia. However, investment in research to develop a chlamydia vaccine could rapidly reduce this impediment [32]. The cost and feasibility of actions to remove impediments would also need to be considered when identifying whether they are cost-effective relative to other actions.

In addition to limiting the extent to which we can achieve recovery targets, impediments to recovery action success also have implications for the optimal allocation of resources. We found that, for target growth rates below around 0.97, the optimal strategy with and without impediments differ little, with reducing vehicle collisions being the preferred strategy. However, for target growth rates between 0.97 and 0.99, the optimal strategies were quite different. Here, in the presence of impediments, investment in habitat restoration rapidly became the preferred strategy, while, with no impediments, investment in reducing vehicle collisions was the preferred strategy. Consequently, it is critical that we consider the implications of impediments for determining the optimal investment of conservation resources among multiple actions. However, whether incorporating information about impediments will change the optimal decision depends on the conservation objectives and targets.

Our analysis demands information that will always be uncertain. However, we were able to quantify uncertainty in achieving the target growth rate due to uncertainty in the population model parameters. Yet, for the return-on-investment curves, we were unable to explicitly estimate levels of uncertainty. Despite the level of uncertainty in the absolute return-on investment-curves being unknown, we believe that the relative cost-efficiencies of each action are likely to be robust. Nonetheless, dealing with such uncertainty in economic costs remain an important area for future research, as costs play a significant role in driving the optimal allocation of resources [33]. To incorporate uncertainty in estimates of cost, where we often do not have good estimates of the level uncertainty, future studies could use approaches such as info-gap decision theory to assess robustness to uncertainty [34]–[37].

An important issue that we did not consider is the presence of time lags between conducting restoration activities and the benefit to the population. For example, restored habitat may not be usable for koalas for 10–15 years after conducting habitat restoration [38]. Baxter *et al.* [39] found that incorporating a lag in the benefit of restoration activities, density dependence and other spatial processes for the helmeted honeyeater (*Lichenostomus melanops cassidix*) did not change the preferred management strategy compared to a simpler projection matrix approach. It is unclear to what extent these lags would change the optimal strategy in our study. However, in a dynamic conservation problem it would probably make habitat restoration preferable earlier, to avoid the risk of population extinction or bottlenecks before restored habitat becomes usable [40]. Therefore, understanding the consequences of these types of dynamic processes and time lags for the optimal allocation of resources among multiple recovery actions is an important area for future research.

Resources are always a limiting factor in achieving species recovery. Therefore, it is necessary to identify cost efficient strategies to recover species, often in the face of multiple threats. However, failure to account for the impediments to success when we allocate resources may lead to a failure to achieve the stated management objective and/or result in sub-optimal investment of scarce conservation resources. If a management objective is not achievable, then consideration of alternative strategies to reduce impediments may actually be an essential component of recovery planning.

## Supporting Information

Appendix S1 **Constructing the return-on-investment curves.**
(DOCX)

Appendix S2 **Nonlinear programming and optimization.**
(DOCX)
