# Supplementary material for: Impediments to the Success of Management Actions for Species Recovery
Source: PLoS One. 2014 Apr 3;9(4):e92430. doi: 10.1371/journal.pone.0092430 (PMC3974711; doi:10.1371/journal.pone.0092430)
Supplement: Appendix S1 — Constructing the return-on-investment curves. (DOCX) [file pone.0092430.s001.docx]

**Appendix S1: Constructing the return-on-investment curves**

***Reducing Vehicle Collisions***

We assumed that management strategies to reduce vehicle collisions include the building of overpasses or underpasses and the installation of exclusion or guide fencing ([Semeniuk et al. 2011](#_ENREF_44)). Cost estimates for these actions were obtained for road upgrades to reduce vehicle collisions at nine locations within the Koala Coast from Caneris and Jones ([2004](#_ENREF_8)) and indexed to 2008 using the Consumer Price Index (CPI). In addition, the mean mortality rates due to vehicle collision per year for each location, as well as the entire Koala Coast from 1997 to 2004 were obtained from Preece ([2007b](#_ENREF_40)).

We then fitted an asymptotic curve to the cumulative mortalities and cumulative costs, assuming that investments are made sequentially in locations from the most cost effective to the least cost effective (i.e., the highest potential number of mortalities mitigated per dollar spent first). The curve fitted was

$m=\eta_{car}\left[1-e^{-\frac{x_{car}}{\gamma_{car}}}\right]$, (1)

where *m* is the cumulative number of vehicle collision mortalities; *x_car_* is the cumulative investment in the vehicle collision reduction action in dollars; $\eta_{car}$is the asymptote; and $1/\gamma_{car}$ that measures cost efficiency. Both $\eta_{car}$ and $\gamma_{car}$ were estimated from the data by least squares. Here, we assumed that the marginal gain per dollar spent declines with the level of investment and that the asymptote represents the maximum number of car collision mortalities that could, in theory, be prevented at these locations if the action was perfectly effective. Next, we scaled up the function in equation 1 to the entire Koala Coast and re-expressed the number of mortalities as a proportion of the total number of car collision mortalities to give

$f_{i,2,car}\left(x_{car}\right)=e^{-x_{car}/\beta_{i,2,car}}$, (2)

where $\beta_{i,2,car}=\gamma_{car}M/\eta_{car}$ for all age classes *i* = 1, 2, 3 and *M* = 268.88 is the mean annual number of car collision mortalities across the whole of the Koala Coast between 1997 and 2004 ([Preece 2007a](#_ENREF_39)) . Note here that we assume that the action is perfectly effective by setting $\alpha_{i,2,car}=0$ in equation 2. Investment in the car collision reduction action was also assumed not to affect mortality cause probabilities other than vehicle collision mortality.

However, it is unlikely that this action would be perfectly effective at reducing vehicle collisions given current technological impediments. Although there is no data on the effectiveness of road underpasses / overpasses and fencing for the Koala Coast, Semeniuk et al. (2011) found that, for an upgrade of the Pacific Highway in New South Wales a road underpass and overpass, with exclusion fencing, reduced koala mortality by 77%. Thus, we estimated the return-on-investment curve with technological impediments to success by fixing $\alpha_{i,2,car}=0.23$ to give the return-on-investment curve

$f_{i,2,car}\left(x_{car}\right)=0.23+0.77e^{-x_{car}/\beta_{i,2,car}}$ (3)

***Reducing Dog Attacks***

An important strategy for reducing dog attacks on koalas is to limit the opportunities for contact between domestic dogs and koalas. This can be affected through legislation, education, or incentives to encourage dog owners to exercise greater control and restraint of domestic dogs. We assumed the provision of enclosures for domestic dog owners as a strategy for encouraging the constraint of dogs at night. The Redland City Council (the local government area that covers the majority of the Koala Coast) currently considers this as a possible management alternative (Daniel Carter personal communication).

We constructed a return-on-investment curve that related the cost of this management action to the proportional reduction in dog attack mortality. To construct this curve, we first obtained data on the number of medium sized and large domestic dogs and the mean annual number of recorded dog related koala mortalities between 1997 and 2008 in each suburb in Redland City Council (Redland City Council unpublished data, Department of Environment and Resource Management unpublished data). We ignored small dogs because these were assumed not to be a threat to koalas. Then, we obtained costs for building outdoor enclosures for medium sized dogs (4' x 8' enclosure) and large dogs (10' x 10' enclosure) from Kennel Solutions (<http://www.kennelsolutions.com.au/dog_kennel_kits/premier_kennel_kits.html>). The costs of outdoor enclosures for medium sized and large dogs were AU$1,326.05 and AU$1,784.42 respectively.

To construct the return-on-investment curve, we obtained data on the number of medium sized and large domestic dogs between 1997 and 2008 in each suburb in Redland City Council (Redland City Council unpublished data). We assumed that small dogs are not a threat to koalas and therefore ignored them. Then, we obtained costs of building outdoor enclosures for medium sized dogs (4' x 8' enclosure) and large dogs (10' x 10' enclosure), that are AU$1,326.05 and AU$1,784.42 respectively from Kennel Solutions (<http://www.kennelsolutions.com.au/dog_kennel_kits/premier_kennel_kits.html>). We calculated the total investment required to supply each dog owner in each suburb with an enclosure for each of their dogs by summing the costs of enclosing medium sized dogs multiplied by the number of medium sized dogs in each suburb with the costs of enclosing large dogs multiplied by the number of large dogs in each suburb.

Using the same approach as for the vehicle collision reduction action, we fitted equation 1 to the cumulative number of dog-related mortalities and the cumulative cost of supplying enclosures for each suburb, assuming investments are made sequentially in suburbs from the most cost effective to the least cost effective. This gave the following relationship

$n=\eta_{dog}\left[1-e^{-\frac{x_{dog}}{\gamma_{dog}}}\right]$, (4)

where *n* is the cumulative number of dog related mortalities; *x_dog_* is the cumulative investment in the dog control action in dollars; $\eta_{dog}$is the asymptote; and $1/\gamma_{dog}$ that measures cost efficiency. Both $\eta_{car}$ and $\gamma_{car}$ were estimated from the data by least squares.

This was then scaled up to the entire Koala Coast in the same way as for vehicle collision reduction action. This gave a return-on-investment curve for the proportion of the total number of dog mortalities, assuming there are no impediments to the success of the action, as follows

$f_{i,3,dog}\left(x_{dog}\right)=e^{-x_{dog}/\beta_{i,3,dog}}$, (5)

where $\beta_{i,3,dog}=\gamma_{dog}N/\eta_{dog}$ for all age classes *i* = 1, 2, 3 and *N* = 55.08 is the mean annual dog related mortality rates across the entire Koala Coast between 1997 and 2008 (Department of Environment and Resource Management, unpublished data). Again, here we assume that the action is perfectly effective by setting $\alpha_{i,3,dog}=0$ in equation 5 and that investment in dog control was assumed not to affect mortality causes probabilities other that dog attack mortality.

However, there are likely to be impediments to the success of this action. A study by Clark ([2006](#_ENREF_12)) found that 36% of dog owners in Redland City Council currently keep their dogs outside at night, but 20% of dog owners indicate that nothing will convince them to constrain their dogs. This suggests that only 16 out of every 36 dog owners (44%), who currently keep their dogs outside at night, will likely adopt dog enclosures. Therefore, this social impediment means that we are only likely to be able to reduce dog attack related koala mortality rates by a maximum of 44% regardless of the level of investment. Therefore, the return-on-investment curve for investment in dog mitigation measures with impediments to its success was assumed to be

$f_{i,3,dog}\left(x_{dog}\right)=0.56+0.44e^{-x_{dog}/\beta_{i,3,dog}}$. (6)

***Habitat Restoration***

The cost of habitat restoration was assumed to consist of the unimproved land value, plus the cost of the restoration activity itself. Unimproved land value was included in the cost on the assumption that land under restoration is taken out of economic production and therefore is an opportunity cost of the restoration. Land value is commonly used to estimate this type of opportunity cost under the assumption that it provides an estimate of the economic benefit for productive land uses and that there is little economic benefit from conservation land usage ([Chomitz et al. 2005](#_ENREF_11)). A return-on-investment curve for habitat restoration was developed by simulating the influence of habitat restoration on natural and disease mortality rates (Rhodes et al. 2011).

We mapped the unimproved land value per hectare for the Koala Coast based on 2006 Queensland Valuation and Sales data (<http://www.dnrm.qld.gov.au/property/valuations/products-services>) and indexed this data to 2008 using CPI. Since reserves and protected areas are already used for conservation, we assumed that these had unimproved land values of zero. We then added the cost of restoration of eucalypt forest at AU$7,605 per hectare estimated from Schirmer and Field ([2000](#_ENREF_46)). Then, we estimated the cost of habitat restoration in each suburb in the Koala Coast by simulating restoration of all areas that we deemed available for restoration within each suburb. In the absence of any impediments to success, we assumed that all land in the Koala Coast was available for restoration. We then considered to consequences of impediments imposed by land-use by assuming that urban areas and intensive land use zones are unavailable for restoration.

In constructing the return-on-investment curves we assumed that investment in habitat restoration starts from the most cost-effective suburb to the least cost-effective suburb based on restoration in each suburb influences the population growth rate (i.e., highest increase in growth rate per dollar spent first). Therefore, we simulated both the cost of restoration and the change in growth rate for each suburb and calculated the change in growth rate per dollar spent for each suburb. Based on suburbs ranked from highest to lowest change in growth rate per dollar spent we fitted an asymptotic exponential function to the cumulative change in natural and disease mortality and the cumulative costs of habitat restoration. The functions fitted to these mortality causes were

$f_{i,1,habitat}\left(x_{habitat}\right)=\alpha_{i,1,habitat}+\left(1-\alpha_{i,1,habitat}\right)e^{-x_{habitat}/\beta_{i,1,habitat}}$, (7)

and

$f_{i,4,habitat}\left(x_{habitat}\right)=\alpha_{i,4,habitat}+\left(1-\alpha_{i,4,habitat}\right)e^{-x_{habitat}/\beta_{i,4,habitat}}$. (8)

These functions were first fitted assuming no land-use impediments and then assuming land-use impediments are present. Note that here, in contrast to the dog control and vehicle collision reduction actions we assume that different return-on-investment for each age class *i* = 1, 2, 3 (actions are assumed not to reduce juvenile mortality).
