# Supplementary material for: Impediments to the Success of Management Actions for Species Recovery
Source: PLoS One. 2014 Apr 3;9(4):e92430. doi: 10.1371/journal.pone.0092430 (PMC3974711; doi:10.1371/journal.pone.0092430)
Supplement: Appendix S2 — Nonlinear programming and optimization. (DOCX) [file pone.0092430.s002.docx]

**Appendix S2: Nonlinear programming and optimisation**

Nonlinear programming is used to study a set of problems where it has to be minimised or maximized over a set of nonlinear constraints ([Theodore 2008](#_ENREF_49)). The objective function (eqn 1) for the case study was considered a nonlinear programming problem because the relationship between the population growth rate λ (C_1_, C_2_,….C_n_) and the total cost for each management action is nonlinear. An active set algorithm from nonlinear programming was used to determine the optimal allocation of resources to each management action for the target population growth rate.

An active set algorithm starts with an active set which is any possible set of variables that we are trying to maximize or minimize ([Mordecai 2003](#_ENREF_34)). In this problem, the active set is the investment in reducing vehicle collision, dog attacks and habitat restoration (C1,C2,C3). Then, it calculates the Lagrange multiplier of the active set and removes the constraints that are impossible and have negative Lagrange multipliers ([Mordecai 2003](#_ENREF_34)). Then, it moves on to another active set and repeats this again until it finds the minimum or maximum active set that satisfies the condition specified by the constraints ([Mordecai 2003](#_ENREF_34)). By using the Lagrange multiplier, this algorithm has the added advantage of breaking a nonlinear problem that is difficult to solve into small linear sub problems that can be solved ([Mordecai 2003](#_ENREF_34)).
